# Neural pathways of attitudes toward foreign languages predict academic performance

**DOI:** 10.3389/fpsyg.2023.1181989

**Published:** 2023-07-26

**Authors:** Di Lu, Xin Wang, Yaozhen Wei, Yue Cui, Yapeng Wang

**Affiliations:** ^1^State Key Laboratory of Cognitive Neuroscience and Learning and IDG/McGovern Institute for Brain Research, Beijing Normal University, Beijing, China; ^2^School of Psychological and Cognitive Sciences and Beijing Key Laboratory of Behavior and Mental Health, Peking University, Beijing, China

**Keywords:** foreign language attitudes, academic performance, fMRI, functional connectivity, graph theory

## Abstract

Learning attitude is thought to impact students’ academic achievement and success, but the underlying neurocognitive mechanisms of learning attitudes remain unclear. The purpose of the present study was to investigate the neural markers linked to attitudes toward foreign languages and how they contribute to foreign-language performance. Forty-one Chinese speakers who hold differentiated foreign language (English) attitudes were asked to complete an English semantic judgment task during a functional magnetic resonance imaging (fMRI) experiment. Multimethod brain imaging analyses showed that, compared with the positive attitude group (PAG), the negative attitude group (NAG) showed increased brain activation in the left STG and functional connectivity between the left STG and the right precentral gyrus (PCG), as well as changed functional segregation and integration of brain networks under the English reading task, after controlling for English reading scores. Mediation analysis further revealed that left STG activity and STG-PCG connectivity mediated the relationships between English attitudes and English reading performance. Taken together, these findings suggest that objective neural markers related to subjective foreign language attitudes (FLAs) exist and that attitude-related neural pathways play important roles in determining students’ academic performance. Our findings provide new insights into the neurobiological mechanisms by which attitudes regulate academic performance.

## 1. Introduction

Attitude is generally defined as a person’s evaluation toward a(n) entity, object, target, or subject matter on a negative to positive (or favorable to unfavorable) continuum (Gjicali and Lipnevich, 2021), and it is a critical factor in predicting individual academic achievement (Credé and Kuncel, 2008). Appropriate attitudes are widely believed to maximize ability and consequently optimize results (Gardner, 1985; Anderman and Wolters, 2006; Oroujlou and Vahedi, 2011). At the behavioral level, evidence has shown that academic attitudes are closely related to academic success across domains, such as reading, math, and science (Masgoret and Gardner, 2003; Chen et al., 2018; Demir-Lira et al., 2019; Gjicali and Lipnevich, 2021). Moreover, a positive attitude is usually associated with good academic performance, whereas a negative attitude often correlates with poor academic outcomes (Masgoret and Gardner, 2003; Demir-Lira et al., 2019; Gjicali and Lipnevich, 2021).

For foreign language (or second language, L2) learning, learners’ attitudes also play important roles. Accumulating evidence from cross-sectional studies shows that learners’ attitudes toward foreign languages are closely related to individual foreign language proficiency, achievement, and other performance (Merisuo-Storm, 2007; Oroujlou and Vahedi, 2011). Importantly, a meta-analysis involving 10,489 individuals demonstrated a significant positive correlation between attitudes toward language learning and second language achievement (Masgoret and Gardner, 2003), and evidence from longitudinal studies further confirmed that positive foreign language attitude (FLA) accounts for the most variance in L2 reading comprehension (Kozaki and Ross, 2011; Smith et al., 2017) and the growth of oral proficiency (HernÁNdez, 2010). More importantly, studies of various age groups (e.g., school-aged children and adults) and sociocultural backgrounds (e.g., Western culture and Eastern culture) support this stable correlation between FLA and academic performance (Masgoret and Gardner, 2003), irrespective of the script of the target language (alphabetic or graphic). That is, the closed relationship between FLA and academic performance is age- and culture-independent.

Explanations for these behavioral findings vary. For example, Merisuo-Storm (2007) argued that negative attitudes toward language learning can reduce learners’ motivation and harm language learning, whereas positive attitudes can do the opposite. Similarly, Oroujlou and Vahedi (2011) supposed that students hold general positive attitudes and beliefs that are reflected in positive emotions in learning and greater persistence, whereas the negative attitudes accompanied by passive feelings inhibit students’ interest and determination to perceive knowledge (Oroujlou and Vahedi, 2011).

However, these explanations might simplify the relationships between attitudes and behavior. First, attitude is a psychological tendency that is expressed by evaluating a particular entity with some degree of favor or disfavor (Eagly and Chaiken, 1993), and it includes a cognitive component (learners’ evaluative beliefs), an affective component (learners’ feelings and emotions regarding the object to be learned), and a conative component (learners’ action readiness and behavioral intentions; Fishbein et al., 1977; Sardegna et al., 2018). Second, attitude often intermixes or interacts with other psychological constructs, such as belief (self-efficacy), emotion (anxiety and enjoyment), and motivation (Masgoret and Gardner, 2003; Oroujlou and Vahedi, 2011; Saito et al., 2018; Sardegna et al., 2018). Third, the roles of attitude in regulating attainment might be antecedent, outcome, and mediating or moderating variable(s) (Asakawa and Oller, 1977). In this sense, clarifying the potential interaction mechanisms between FLA and L2 performance purely based on behavioral studies is difficult.

Crucially, despite decades of behavioral studies, the underlying neural pathways that can explain the effects of learning attitudes on learning performance have yet to be identified. To our knowledge, only three studies in the domain of mathematics have explored the neurocognitive mechanisms of math attitudes to date (Chen et al., 2018; Demir-Lira et al., 2019; Suarez-Pellicioni et al., 2021).

In a pioneering study related to the neurocognitive mechanisms of math attitude, Chen et al. (2018) investigated the neural mechanisms underlying the link between positive attitude and academic achievement in 6–11-year-old children who solved single-digit additions. Specifically, they tested competing hypotheses regarding the differential roles of affective-motivational and learning-memory systems and found that a positive attitude was associated with increased hippocampal learning-memory system engagement, but it was not associated with an enhanced response in the amygdala and ventral striatum. Notably, the increased hippocampal response during numerical tasks observed in their study mediated the relationship between positive attitude and efficient problem solving, leading to academic success in children.

In a second study focused on math attitudes, Demir-Lira et al. (2019) investigated the effects of the interaction between math skill and math attitudes on the neurocognitive basis of arithmetic processing (single-digit multiplication) in 8–15-year-old children. They observed that positive math attitudes were correlated with less activation in the left IFG. Moreover, they found that the relationship between math attitudes and the neural basis of multiplication varied depending on math skill. Positive math attitudes were associated with a greater activation of the left IFG only among children with lower math skills. They interpreted the greater left IFG activation as reflecting effort invested in problem solving.

In a third study of math attitudes, Suarez-Pellicioni et al. (2021) longitudinally followed some of the participants in Demir-Lira et al. (2019) study to examine the neurocognitive mechanisms underlying math attitudes and math improvement. They found that for improvers, more positive math attitudes were related to greater left IFG activation, but this effect was not identified in nonimprovers. They proposed that greater left IFG activation was associated with the investment of effort and represented the neurocognitive mechanisms by which positive math attitudes lead to improvement in multiplication skill over time. Taken together, these findings suggest that learning attitudes might function by modulating the activation of domain-general learning-memory systems or effort-related brain regions during mathematical processing. Although these studies of math attitudes provide some insights for understanding the neurocognitive mechanisms of academic attitudes, no study has directly investigated the neural basis related to foreign language attitudes. Unlike math attitudes, attitudes toward foreign language might be more complex and are related to a learner’s preferences for the subject (foreign language or L2) or the associated culture (Wright, 2006; Sakuragi, 2008).

The current study aimed to examine the underlying neural markers and pathways of FLA and how they contribute to language performance during a foreign language (English) reading task. To investigate these questions, we used functional magnetic resonance imaging (fMRI) to study a sample of Chinese college students who learned English as a foreign language (EFL) when they performed an English semantic judgment task. It is well known that both L1 and L2 (foreign language) reading recruited the dorsal and ventral networks (Oliver et al., 2017; Verhoeven et al., 2019). The dorsal network includes the parietal lobe, superior temporal gyrus (STG), and inferior frontal gyrus (IFG), and the ventral one includes the occipital-temporal (vOT) and anterior IFG regions. The former is thought to subserve phonological processing, and the latter supports mapping of orthographic-lexical stimuli onto semantic representations (Oliver et al., 2017). To explore neural markers of FLA, we first applied brain activation and seed-based functional connectivity analyses to investigate differences between students with positive and negative FLA after controlling for behavioral performance. We then further employed a complex brain network analysis based on graph theory to characterize topological differences between the two groups (Bullmore and Sporns, 2009, 2012). If potential neural markers related to FLA were identified, we expected to observe differences in brain activation and functional connectivity between the two groups. At the whole-brain network level, we also expected that positive attitudes might enhance brain network efficiency during foreign language processing.

To examine the potential neural pathways by which FLA contribute to language performance, we further conducted a mediation analysis to identify whether brain activation and functional connectivity mediate the relationship between attitudes and foreign language performance. Previous work on math attitudes demonstrated that the effects between positive math attitudes and math achievement are mediated by memory strategy and greater hippocampal activation (Chen et al., 2018). Therefore, we expected that the brain’s activation and functional connectivity also constitute the link between attitudes and foreign language achievement. Exploring the neural substrates of FLA can not only help us determine attitude-related effects in the specific domain but also expand our understanding of the domain-general or domain-specific mechanisms of learning attitudes This exploration will provide important insights for understanding the fundamental mechanisms of attitudes toward foreign languages and their association with language achievement and other performances, which might help us develop proper interventions to increase the efficiency of foreign language teaching and inspire learners’ potentials.

## 2. Methods

### 2.1. Participants

Forty-one college students (20 females, average age = 18.46 ± 0.75 years) were enrolled in the study. All participants were native speakers of Chinese and began to learn English as a second language starting in the first grade of primary school (age of acquisition = 6.02 ± 1.59 years). They all came from Beijing and had highly similar second language education backgrounds. All participants were healthy, right-handed and had normal or corrected-to-normal vision (Yuan et al., 2021; Li et al., 2023). All participants signed an informed consent form before the experiment, which was approved by the Institutional Review Board of Beijing Normal University.

### 2.2. Behavioral tests

#### 2.2.1. Foreign language attitudes

To qualify the participants’ attitudes toward foreign languages, we used the Attitudes Toward English Learning Scale (ATELS; Pae and Shin, 2011), an eight-item self-assessment questionnaire aimed to measure learners’ attitudes toward a foreign language (e.g., I truly enjoy learning English). The participants were asked to evaluate how much they agreed or disagreed with each item using a five-point scale (from 1-strongly disagree to 5-strongly agree). The Cronbach’s alpha of the scale is 0.87. The total score of the ATELS was regarded as an indicator of learners’ FLA, and all participants were divided into the positive FLA group and negative group based on the median ATELS. The two groups did not differ significantly in age, sex, IQ, age of acquisition, or L1 proficiency (see Table 1).

**Table 1:** Demographics and task performance of the two groups.

	Positive group	Negative group	*p* value
*N*	19	22	/
Gender (male/female)	9/10	12/10	/
Age (years)	18.21 ± 0.42	18.68 ± 0.89	0.065
Raven score	56.05 ± 2.30	56.50 ± 2.35	0.401
AoA	5.79 ± 1.48	6.23 ± 1.69	0.39
English attitude	32.42 ± 3.89	23.77 ± 2.72	< 0.001^***^
Chinese reading score	50.74 ± 10.18	44.50 ± 10.84	0.06
English reading score	81.42 ± 16.74	62.82 ± 20.78	0.003^**^
Semantic task_ACC(%)	0.66 ± 0.13	0.53 ± 0.15	0.006^**^
Semantic task_RT(ms)	1022.95 ± 59.07	1027.41 ± 73.14	0.83

*N*, number of participants; AoA, age of acquisition for the second language; ACC, accuracy; RT, response time. ^**^p < 0.01, ^***^p < 0.001.

#### 2.2.2. Reading fluency test

English reading performance was assessed using the reading fluency test (RFT) of the Woodcock Johnson-III (Woodcock et al., 2001), which has been widely used to probe English reading fluency and ability in previous studies (August et al., 2006; Francis et al., 2006). The test consists of 98 items that evaluate learners’ general English reading ability, especially reading fluency (e.g., you can eat an apple). The participants were asked to judge whether the meaning of each English sentence was reasonable, and the total RFT score was used as the indicator of a learner’s reading fluency (see Table 1 for more details on demographics and behavioral performances).

### 2.3. fMRI experimental procedure

The participants performed an English semantic judgment task in the scanner, in which they were asked to decide whether two visually presented English words were semantically related or not. All words were 4–6 letters long (mean = 4.4). An arrow direction judgment task was used as a control task, in which the participants were asked to judge whether the arrow was pointing upward or downward, and both the experimental task and the control task were successfully used in a previous study (Tan et al., 2011). A block design was used, in which the semantic task was alternated with the baseline task (arrow direction judgment). Each experimental block consisted of 12 trials, whereas each baseline block consisted of eight trials. In each trial, stimuli (word pairs or an arrow) in white were displayed on a black background for 1,500 ms, followed by a 500 ms fixation interval. The participants were instructed to press a yes button for semantically related word pairs (or an upward arrow) using their right index finger or press a no button with the right middle finger for semantically unrelated word pairs (or a downward arrow). Half of the word pairs were semantically related, and half were not. The participants were asked to perform the task as quickly and accurately as possible.

### 2.4. MRI acquisition

All images were acquired using a 3 T Siemens Trio Scanner at Beijing Normal University. An echo planar imaging (EPI) sequence was used for functional imaging with the following parameters: TR = 2,500 ms, TE = 30 ms, flip angle = 90°, and scan order = interleaved. Matrix size = 64 × 64, slice thickness = 3 mm, and voxel size = 3 mm × 3 mm × 3 mm. Additionally, a high-resolution T1-weighted 3D image (MPRAGE) was acquired with the following parameters: TR = 2,530 ms, TE = 3.39 ms, flip = 7°, matrix size = 256 × 256, slice thickness = 1 mm, and voxel size = 1 mm × 1 mm × 1 mm.

### 2.5. fMRI data analysis

#### 2.5.1. Whole-brain activation analysis

SPM 12 was used for image preprocessing and statistical analysis.^1^ Functional images were first corrected for slice acquisition delays and realigned to the first image of the first run to correct for head movements. The images were further spatially realigned and coregistered to their corresponding anatomical images. The resultant images were then spatially normalized to the Montreal Neurological Institute (MNI) space. After normalization, all images were resampled into 3 mm × 3 mm × 3 mm voxel sizes and further spatially smoothed using a Gaussian kernel with 8 mm full width at half maximum (FWHM). An individual participant’s activation t map was generated using the general linear model, in which time series were convolved with the canonical hemodynamic response function and were high-pass-filtered at 128 s.

The individual contrast images of the semantic judgment minus arrow judgment were computed as a first-level analysis, and the contrast maps were then subjected to a second-level analysis to compare activation differences between the positive and negative groups by performing two-sample *t* tests. An FWE-corrected cluster-level threshold of *p* = 0.05 (defined using a voxel-level threshold of *p* = 0.001) was applied to all whole-brain statistical maps to assess brain activations.

#### 2.5.2. Functional connectivity analysis

We performed seed-to-voxel analysis to identify differences in the functional connectivity among the clusters identified through the activation analysis and other regions between the positive FLA group and the negative group. To this end, seed ROIs were created using the clusters that were significantly related to FLA. Using the DPABI toolbox v4.2 (http://rfmri.org/dpabi; Yan et al., 2016), we first averaged the time series of all voxels in each seed. We then temporally correlated the seed ROIs and all the other voxels in the brain, and participant-level correlation maps were obtained. For standardization purposes, the correlation maps were normalized to *z* maps. At the group level, we conducted a two-sample *t* test between group *z* maps to detect the association between FC and FLA, with English reading score as a controlling variable. Functional connectivity maps survived a corrected cluster-level threshold of *p* < 0.001 (single voxel *p* < 0.001, and a minimum cluster size of 50 voxels) using the Gaussian random field approach (Worsley et al., 1992).

#### 2.5.3. Graph theoretical analysis

##### 2.5.3.1. Network construction

The graph theoretical analysis was performed using the GRETNA toolbox (graph theoretical network analysis: http://www.nitrc.org/projects/gretna; Wang et al., 2015). Based on the automated anatomical labeling (AAL) atlas with 90 ROIs, we extracted the time series for each AAL ROI by calculating the mean (across voxels) signal for each time point, and a 90 × 90 Pearson correlation matrix was created for each participant for the semantic judgment condition. We constructed binary undirected functional networks using a sparsity threshold (5% ≤ sparsity ≤50%, interval = 5%) to comprehensively estimate topological properties covering a wide range of sparsity and remove spurious edges as much as possible.

##### 2.5.3.2. Network properties and group comparisons

We calculated graph properties characterizing the global-level network organization for each participant, including the following: (1) functional segregation, which is the ability for specialized processing within densely interconnected groups of brain regions, including the metrics of local efficiency and clustering coefficient (Bullmore and Sporns, 2009, 2012); (2) functional integration, which refers to the capacity of the network to rapidly combine specialized information from distributed brain regions and includes the metrics of characteristic path length and global efficiency (Bullmore and Sporns, 2009, 2012); and (3) small-worldness, which reflects an optimal balance of functional integration and segregation (Bullmore and Sporns, 2009, 2012). To examine the group differences of all the network metrics mentioned above, ANCOVA was used for between-subject comparisons and regressed-out covariates of English reading fluency. To correct for multiple comparisons, we used a Bonferroni corrected threshold at a significance level of 0.05.

#### 2.5.4. Brain-behavior mediation analysis

For brain activation and functional connectivity showing a significant association with FLA, we used mediation analysis to examine whether neural correlates of FLA mediate the association between behavioral FLA and English reading performance. Mediation analysis was conducted using the PROCESS macro in SPSS (Hayes, 2013).

During mediation analysis, FLA and English reading fluency were defined as the independent (predictor) variable and dependent (outcome) variable, respectively. We defined the mediator variables based on the brain statistical maps resulting from the group differences in activation and seed-based connectivity analysis described above. The significance of the indirect effect was determined using a bootstrapping method with 5,000 iterations. If a 95% confidence interval (CI) did not contain zero, then the indirect effect was significant (Preacher and Kelley, 2011; Hayes, 2013).

## 3. Results

### 3.1. Behavioral results: FLA predicted foreign language performance

To reveal the relationships between FLA and English reading performance, we correlated individuals’ FLAs with English reading scores. The results showed a significant positive correlation between FLA and English reading proficiency (fluency; *r* = 0.34, *p* < 0.001). Critically, the association between FLA and language performance remained significant after adjusting for age and IQ in a multiple regression analysis.

### 3.2. fMRI results

#### 3.2.1. FLA-related activation differences

**Figure 1:**
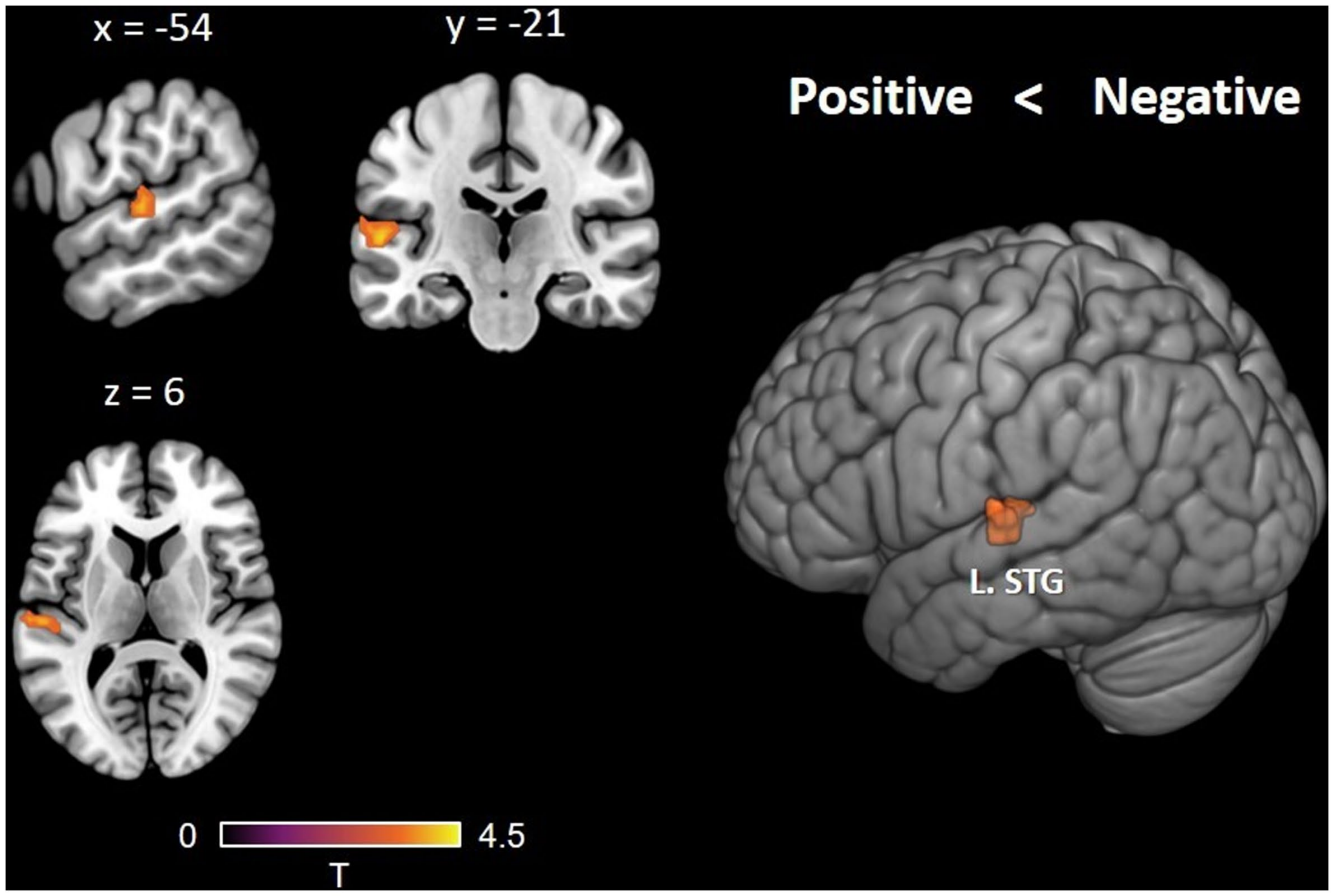
Brain activation differences between the PAG and NAG in the English semantic judgment task. After controlling for English reading fluency, increased activation in the left STG was observed when comparing the NAG with the PAG.

First, we performed a univariate analysis to investigate group differences during English semantic decisions. After controlling for English reading scores, the two-sample *t* test of the whole-brain analysis revealed that, compared with the positive attitude group (PAG), the negative attitude group (NAG) showed increased activation in the left STG (BA 48, MNI: −54, −21, 6; *p* < 0.05, clusterwise FWE corrected; cluster size = 40 voxels). Relative to NAG, we failed to find stronger brain activation in the PAG (Figure 1).

#### 3.2.2. FLA-related functional connectivity differences

**Figure 2:**
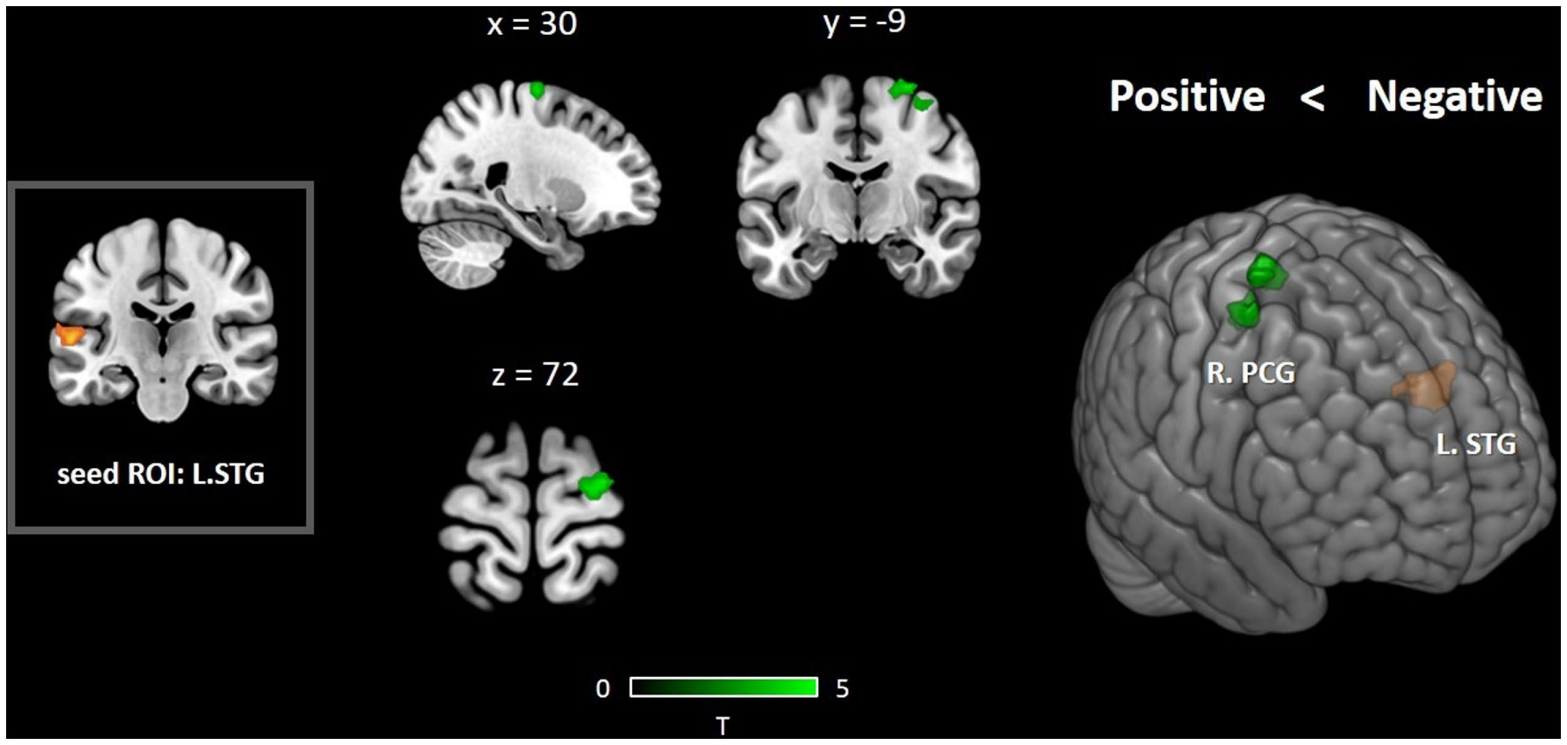
Seed-based functional connectivity differences between the PAG and NAG in the English semantic judgment task. After controlling for English reading fluency, increased FC between the left STG and right PCG was observed when comparing the NAG with the PAG.

Since a significant between-group activity difference was identified in the left STG, the left STG was taken as a seed region to compare the seed-to-voxel functional connectivity differences between PAG and NAG, with English reading scores as the nuisance covariate (*p* < 0.001, GRF corrected). The results showed that the NAG exhibited significantly stronger functional connectivity between the left STG and right precentral gyrus (PCG) than the PAG. For the opposite comparison, we did not observe any difference in functional connectivity between the two groups (Figure 2).

#### 3.2.3. FLA-related topological properties

**Figure 3:**
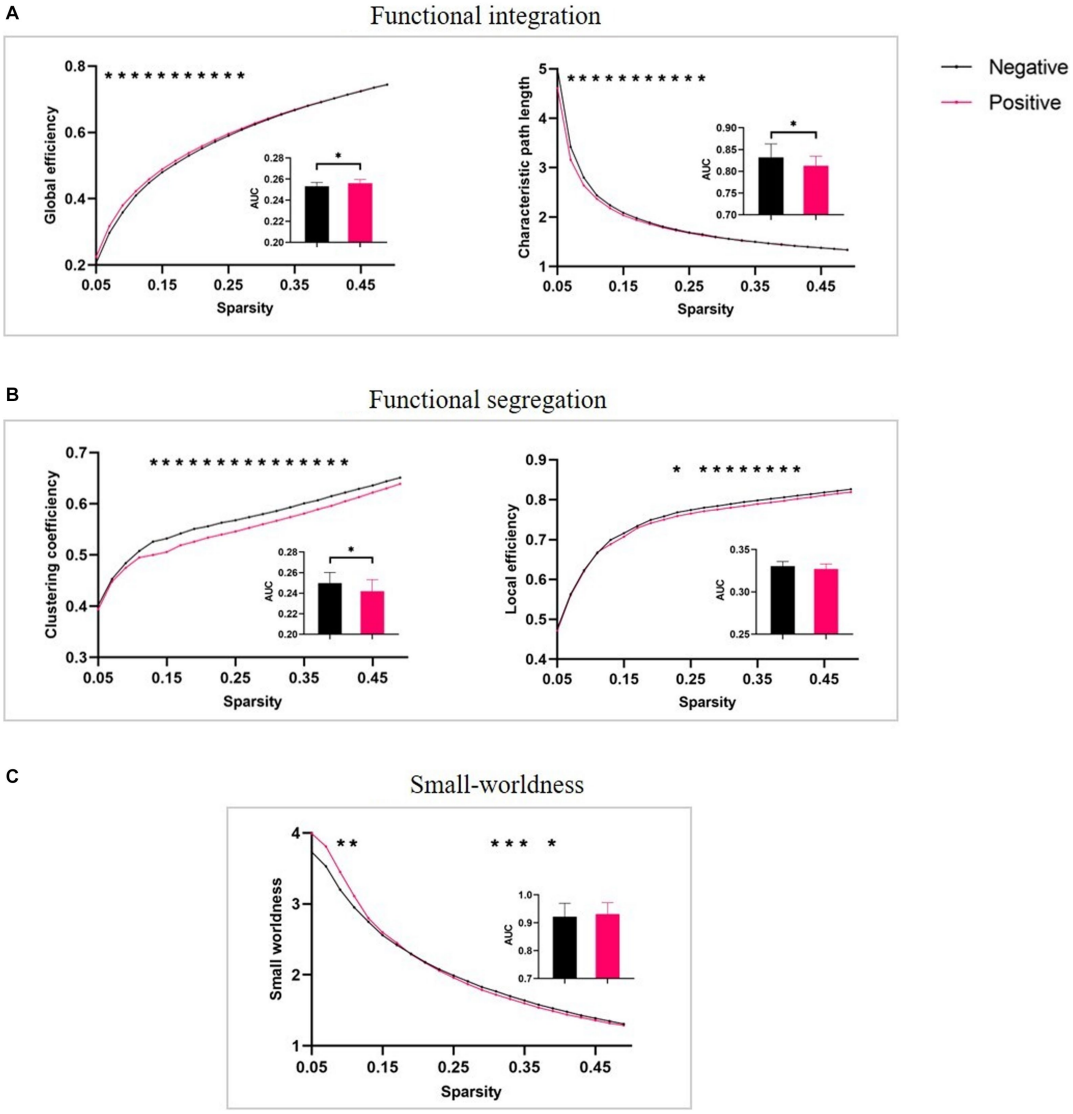
Between-group comparisons in graph properties of functional networks. **(A)** functional integration: global efficiency (E_glob_) and characteristic path length (L_p_), **(B)** functional segregation: clustering coefficiency (C_p_) and local efficiency (E_loc_), and **(C)** small-worldness. Inset maps (with mean and standard error) show significant group effects of the area under the curve (AUC) in E_glob_, L_p_, and C_p_, *p* < 0.05.

To explore FLA-related topological properties, we applied graph theoretical analysis to test whether topological properties during the English semantic task can distinguish the PAG from the NAG. The results showed significant group differences in network integration and segregation at the sparsity-integrated level. Specifically, the network engaged by the positive group exhibited significantly higher global efficiency (for 0.05 < T < 0.15 and 0.4 < T < 0.5) but lower characteristic path length (for 0.05 < T < 0.2 and 0.4 < T < 0.5) and clustering coefficiency (for 0.5 < T < 0.5) than that engaged by the negative group. For the network local efficiencies and small-worldness, we failed to find any difference between the two groups (see Figure 3 for a summary of these findings).

#### 3.2.4. Brain-behavior relationships

To reveal the brain-behavior relationship, we applied mediation analysis to examine whether the relationships between FLA and foreign language performance could be explained by attitude-related brain activity and functional connectivity.

**Figure 4:**
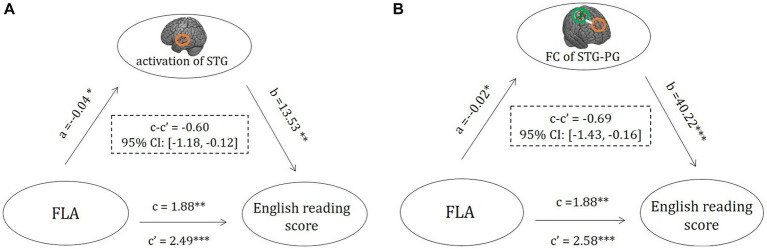
The potential pathways of FLA-related neural markers that mediate FLA and foreign language performance. **(A)** Pathways of left STG activity that mediate FLA and foreign language performance (reading fluency) and **(B)** pathways of FC of STG-PCG that mediate FLA and foreign language performance (reading fluency). FC, functional connectivity; PCG, precentral gyrus. ^*^p < 0.05, ^**^p < 0.01, ^***^p < 0.001.

At the activity level, adding activation in the left STG as a mediator showed that left STG activation significantly and indirectly mediated the relationship between FLA and foreign language reading performance (see Figure 4A; indirect effect = −0.60, 95% CI = [−1.18, −0.12], *p* < 0.05).

At the connectivity level, adding FC of the left STG and right PCG as a mediator showed that the association between FLA and reading performance was mediated by FC (see Figure 4B; indirect effect = −0.69, 95% CI = [−1.43, −0.16], *p* < 0.05). Taken together, our findings indicated that the FLA influenced foreign language performance through task-related brain activity and connectivity.

## 4. Discussion

In the present study, we first used task-based fMRI to investigate the neurobiological correlates of FLA from brain activation, functional connectivity, and large-scale brain network levels and the roles of FLA-related brain activity and connectivity in connecting FLA and foreign language achievement. Overall, our study identified the neural markers of the FLA and the neural paths of the FLA that influence foreign language learning and achievement.

### 4.1. Brain activity markers differentiating PAG from NAG

At the whole-brain level, we found that NAG showed enhanced activation in the left STG in the English semantic judgment task compared to the PAG.

The left STG is generally considered a core brain region in language function, and it is primarily involved in auditory processing and speech comprehension (Gernsbacher and Kaschak, 2003; Martin, 2003). Importantly, the left STG and adjacent gyral regions have repeatedly been related to audiovisual print–speech integration, especially grapho-phonological conversion (Blau et al., 2009; Kronschnabel et al., 2014; Ye et al., 2017). In addition, previous studies found that this area played important roles in integrating phonological decoding and semantic information to facilitate semantic access in the process of English word reading (Hu et al., 2010). For example, increased left STG activation has been observed when bilingual participants performed English semantic tasks (Tan et al., 2005). In other words, the left STG plays important roles in both audiovisual print–speech integration and phonology-semantics integration. More importantly, as an important region of the core language system, the activation in the left STG was supramodal or modality independent and showed shared cortical activation across spoken, written and signed languages in Dutch speakers, Chinese monolinguals, and Chinese speech-sign bilinguals (Liu et al., 2020).

Generally, brain systems that involve affect, motivation, learning, and memory have been hypothesized to underpin the influence of positive attitudes on academic learning and achievement (Chen et al., 2018). Indeed, Chen et al. (2018) studied math attitudes by employing a single-digit-addition task and found that a positive attitude was associated with increased engagement of the MTL learning-memory system (bilateral hippocampus) but not the affective-motivational system (amygdala or ventral striatum).

In addition to the hippocampal memory system, previous studies on math attitudes also reported that math attitudes correlated with activation in the left IFG when the participants performed a single-digit multiplication task, but this attitude-related IFG activity was observed only for children with positive math attitude but low math skill, and they argued that IFG activity might reflect controlled effort and the retrieval of multiplication facts (Chen et al., 2018; Demir-Lira et al., 2019; Suarez-Pellicioni et al., 2021).

In our dataset, we only observed attitude-related activation in the NAG, and this finding is generally consistent with Demir-Lira’s results. In their study, they observed that positive attitudes toward math correlated with less activation in the left IFG. With respect to activity intensity (e.g., increased or decreased), the neural function of academic attitudes seems to be partly domain independent. Since the current study employed different tasks from previous studies (e.g., Chen et al., 2018), it is likely that the different observations are process-driven instead of domain-driven. It is worth mentioning that these two driven might be intermixed and hard to separate from each other. Based on evidence from math attitudes, the larger involvement of the left STG might indicate that negative learners require more effort to recruit phonological processing and semantic integration during English word reading and facilitate task performance.

### 4.2. Functional connectivity markers differentiating PAG from NAG

In addition to differentiating the PAG from the NAG, the activity of the left STG also differed in terms of functional connectivity (FC). Specifically, the seed-based correlation analysis revealed that the FC between the left STG and right precentral gyrus (PCG) was stronger in the negative group than in the positive group. The left PCG has been well documented to be implicated in many functional MRI studies of language and reading (Dehaene et al., 2001; Yen et al., 2019), and some studies related the right PCG to higher-order cognitive mechanisms, such as language production and comprehension (Dickens et al., 2019). Specifically, the right PCG was activated during phonetic planning and concrete semantic representations (Papeo et al., 2015), and it played an important role in sound-motor integration during word generation (Alario et al., 2006). In addition, the right PCG has been reported to be one of the crucial regions for bilingual language control (Luk et al., 2012), and connectome analysis found that early Japanese-English bilinguals showed dense connectivity between the right putamen and PCG compared to Japanese monolinguals and late bilinguals (Mitsuhashi et al., 2020).

Because the left STG is also related to a variety of language processing, the reinforced STG-PCG connectivity in the negative learners may reflect increased investment in reading-related cognitive resources. Furthermore, the negative learners exhibited reinforced FC between the left and right hemispheres to improve their performance during semantic decision-making. Indeed, a previous study supported this possibility and showed that interhemispheric functional brain connectivity could predict new language learning success in adults (Sander et al., 2022). In short, although the role(s) of the right PCG in foreign language attitudes remains unclear, we speculate that the FC between the left STG and right PCG plays a critical role in maintaining reading performance, especially for negative learners.

### 4.3. The topological properties of the large-size brain network differentiating PAG from NAG

To reveal brain network properties that differentiate the PAG from the NAG, we compared the network topology between the positive and negative learners using graph theory analysis.

The results showed that the positive group displayed significantly higher global efficiency (Eg) and shorter characteristic path length (Lp) in the whole-brain network than the negative group, suggesting that positive learners have more efficient and extensive neural pathways to bring them an advantage in network integration capability (Achard and Bullmore, 2007; Rubinov and Sporns, 2010). In contrast to the PAG, the NAG showed an increased clustering coefficient (Cp), which indicates a greater tendency for functional segregation and the formation of clustered connections (Bullmore and Sporns, 2012).

Although the relevance between network topology properties and academic attitudes has not yet been established, evidence from other domains showed that a brain network with intensifying integration and weakening segregation was associated with cognitive advantages. For example, compared with L2 reading, L1 reading recruited a more globally efficient but less clustered functional network topology, which represents more optimized functional network organization during L1 processing (Feng et al., 2015), and individuals with more active moods and less anxiety have larger global efficiency and shorter path length during tasks (Park et al., 2014, 2016). In addition, evidence from short-term language training suggested that less segregation (smaller clustering coefficient) was associated with successful language learning (Sheppard et al., 2012; Yang et al., 2015), and children with L2 reading impairment exhibited higher local network efficiency (Liu et al., 2016). In the context of this study, English semantic judgment is a complex cognitive process that requires the interactive collaboration of several brain networks involved in orthographic, phonological, and semantic processing (Xu et al., 2005; Binder et al., 2009; Friederici et al., 2009; Price, 2012). Therefore, positive learners likely could easily and flexibly use long-range neural pathways to integrate whole-brain resources, and this coherent and cost-efficient network organization could help them more efficiently complete the foreign language task. Notably, we could not infer causal relationships between FLA and brain network properties in the present cross-sectional study. In this sense, future studies should explore this issue based on longitudinal designs.

### 4.4. The neural pathways connecting FLA with academic performance

Behaviorally, stable associations between FLA and foreign language achievement have been repeatedly reported in previous investigations (Masgoret and Gardner, 2003; Zhang et al., 2020; Papi and Khajavy, 2021). What are the potential neural pathways underlying these associations? To answer this question, we performed brain-behavior mediation analysis. Our results showed the critical roles of left STG activation and STG-PCG functional connectivity in mediating the relationship between FLA and foreign language performance, and these findings provide important insights for understanding the roles of FLA-related brain activation and FC in foreign language processing and learning. Since the left STG and right PCG are important regions for lexical-semantic processing (Tan et al., 2005; Hart et al., 2012), and the left STG plays a hub-like role in successful second language learning (Yang et al., 2015), we speculate that negative attitudes related to STG hyperactivation and intensive STG-PCG connectivity might reflect a higher effort or requirement for lexical-semantic processing in the NAG to compensate for the global inefficiency of the brain network and further promote in-scanner foreign language performance, and the results from mediation analysis supported this possibility. Although negative correlations were identified between FLA and left STG activation and STG-PCG connectivity, positive correlations were revealed between reading performance and brain activation as well as functional connectivity. These findings suggest that although we failed to find attitude-specific activity in the learning-memory system (e.g., hippocampus) or emotion-motivation system (e.g., amygdala or ventral striatum), academic attitudes might exert their effect through task-related brain regions (e.g., STG) or networks (e.g., STP-PCG connectivity). In summary, our study indicated that left STG activity and STG-PCG connectivity might be potential neural pathways that explain the impact of FLA on foreign language achievement. More specifically, the FLA might affect STG activity and functional connectivity and further influence individual academic performance.

It is worth mentioning that the sample size of the present study is relatively small, which may increase the probability of false positive effects (Ioannidis, 2005) and lead to low power (Ioannidis, 2005; Bossier et al., 2020). Future studies with a larger sample size or longitudinal design could deepen our understanding of the mechanism of FLA.

## 5. Conclusion

In conclusion, the current study demonstrated, for the first time, that subjective academic attitudes have objective neural signatures and can reshape individuals’ brain activities in the task state. The FLA is associated with changed activity of the left STG, FC of the STG-PCG, and the topological properties of the brain network during the English reading task. Since the STG and PCG play important roles in language and reading, these findings imply that FLA-related neural signatures might not rely on the learning-memory system or emotion-motivation system but depend on task-related brain regions or networks. Importantly, compared with existing studies of math attitudes, the neural signatures of academic attitudes seem to be domain specific. More importantly, academic attitude-related neural predictors underlie the potential pathways that contribute to individuals’ foreign language performance.

## Data availability statement

The raw data supporting the conclusions of this article will be made available by the authors, without undue reservation.

## Ethics statement

The studies involving human participants were reviewed and approved by the Institutional Review Board of Beijing Normal University. The patients/participants provided their written informed consent to participate in this study.

## Author contributions

YPW and DL designed the study. DL and XW analyzed the data, drew the figures, and wrote the manuscript. YPW checked and,revised and edited the manuscript. YZW, YC, and DL performed the experiments. All authors contributed to the article and approved the submitted version.

## Funding

This study has been supported by the Scientific and Technological Innovation 2030 - the major project of the Brain Science and Brain-Inspired Intelligence Technology (2021ZD0200500).

## Conflict of interest

The authors declare that the research was conducted in the absence of any commercial or financial relationships that could be construed as a potential conflict of interest.

## Publisher’s note

All claims expressed in this article are solely those of the authors and do not necessarily represent those of their affiliated organizations, or those of the publisher, the editors and the reviewers. Any product that may be evaluated in this article, or claim that may be made by its manufacturer, is not guaranteed or endorsed by the publisher.

## Footnotes

^1^ http://www.fil.ion.ucl.ac.uk/spm
